# Preparation of quasi-core/shell structured composite energetic materials to improve combustion performance†

**DOI:** 10.1039/d3ra02732e

**Published:** 2023-06-13

**Authors:** Ruihao Wang, Lanting Yang, Zhenwei Zhang, Wenkui Song, Dunju Wang, Changping Guo

**Affiliations:** a Co-Innovation Center for New Energetic Materials, Southwest University of Science and Technology Mianyang 621010 PR China ruihao0847@163.com

## Abstract

Composite explosives with fast reaction rate, high energy release efficiency, and remarkable combustion performance can be obtained by the interaction between homogeneous energetic materials and heterogeneous energetic materials and have broad application prospects. However, ordinary physical mixtures can easily cause separation between the components in the preparation process, which is not conducive to reflecting the advantages of composite materials. In this study, high-energy composite structured explosives with RDX modified by polydopamine as the core and PTFE/Al as the shell were prepared using a simple ultrasonic method. The study of morphology, thermal decomposition, heat release, and combustion performance demonstrated that the quasi-core/shell structured samples have higher exothermic energy, faster combustion rate, more stable combustion characteristics, and lower mechanical sensitivity than the physical mixture.

## Abstract 2

The pRDX@PTFE–Al quasi-core/shell structured high-energy explosives were prepared and found to possess superior combustion and mechanical sensitivity properties.

## Graphical abstract

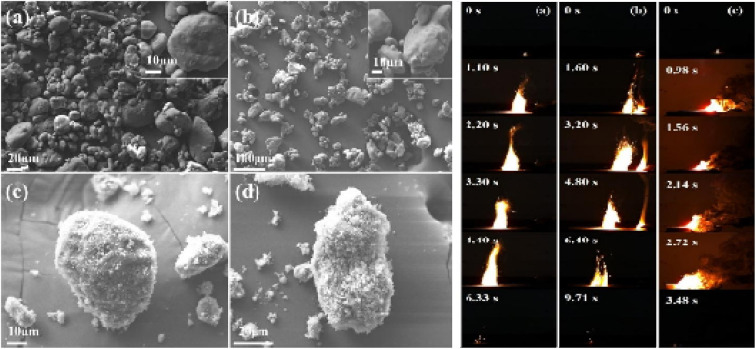

## 1. Introduction

Energetic materials can generally be divided into heterogeneous energetic materials with high energy density (*e.g.*, thermite) and conventional homogeneous energetic materials (*e.g.*, RDX).^1^ Heterogeneous energetic materials tend to maximize the energy density by changing the ratio between the oxidant and fuel to reach a complete balance, and the reaction kinetics between the two substances is controlled by mass transfer and thermal diffusion. Nevertheless, their energy release rate is lower than the corresponding value obtained by the molecular decomposition chemical dynamics processes for homogeneous energetic materials due to the physical separation between the two substances.^2,3^ The energy density of homogeneous energetic materials can only reach half that of heterogeneous energetic materials, while the high reaction rate and gas production rate of homogeneous energetic materials increase the energy output efficiency.^4^

Nano-thermite (metastable intermixed composite, MIC) is a typical heterogeneous energetic material, mainly composed of nano-scale metal fuels and metal oxides. It has a lower reaction temperature, high combustion speed, microscale self-propagation, and other characteristics.^5–7^ The key conditions for heat transfer are a large number of gas products and high pressure, and the MIC combustion process has fewer gas products, leading to limited further application.^8^ Moreover, homogeneous energetic materials have advantages in generating high-pressure gas during combustion or detonation.

Unfortunately, its combustion velocity is generally low. RDX, as the most widely used explosive, has a detonation velocity of over 8000 m s^−1^, with a burning velocity of less than 10 m s^−1^. Therefore, the combination between homogeneous energetic materials and heterogeneous energetic materials would be a complementary strategy to improve the performance of the overall energy system. During the combustion process, the addition of homogeneous energetic materials can chemically interact with nano-aluminum to generate high pressure; the heterogeneous material with nano-aluminum as the main component provides can curtail reactants and improve reaction activity for the composite system.^9,10^ Moreover, many researchers have explored and verified the synergy of the two energetic materials. The flame tube measurement results of RDX@Fe_2_O_3_–Al demonstrate that the composite material is less sensitive to impact, friction, and electric sparks. RDX, as the core, can be burned by the thermal combustion of the thermite. Simultaneously, the high pressure and gas generated by the RDX combustion accelerate the shell's mass transfer and thermal diffusion, accelerating the transition from combustion to detonation.^6^ Furthermore, the thermal and combustion properties of Al/CuO/PVDF/RDX composite microspheres have been significantly improved, with shorter delay time and higher pressure. Combustion experiments reveal that the combustion performance of composite microspheres is majorly affected by the content of RDX.^11^ These results suggest an excellent synergistic effect between RDX and nano-thermite. The large amount of gas generated by the decomposition of explosives can make up for nano-thermite pressure loss during combustion. The pressure generated in the reaction zone increases the gaseous product in the future. Besides, diffusion of the void volume in the reaction zone enhances mass and heat transfer efficiency, as well as the composite material's superiority.^12^

The thermite in the above studies all consisted of metal oxides and metal fuels, and the thermodynamic advantages of metals are generally offset by their relatively long ignition delay, low combustion rate, and condensation products such as oxides formed after combustion.^13^ The use of fluoropolymers instead of metal oxides is a good alternative, and aluminum can react with fluoropolymers to produce more easily sublimated aluminum fluoride (AlF_3_) rather than refractory alumina oxide (Al_2_O_3_). Therefore, more gas products can be produced by using fluoropolymers instead of oxidizers for metal fuels, so as to avoid or reduce the two-phase loss of metal oxide coalescence formation with relatively little impact on energy release or flame temperature. Attributed to its high energy density (21 kJ cm^−3^) and its practical application in propellants, heterogeneous explosives, and pyrotechnics, the reaction material is composed of fuel (Al), and polytetrafluoroethylene (PTFE) has attracted wide attention.^14^ The surface of nano-aluminum contains 10–25 wt% alumina. During the reaction, the active aluminum needs to break the shell of inert alumina, whereas the high melting point of alumina makes the diffusion of reactants relatively slow, resulting in a low energy output;^15^ at a higher heating rate, PTFE can remove the oxide layer on the surface of nano-aluminum and increase the direct contact area between oxygen and aluminum, contributing to the increased reaction rate.^16,17^ The study found 70% Al and 30% PTFE can reach the aluminum fluoride sublimation temperature.^18^ Simultaneously, PTFE has low friction, high-temperature resistance, and excellent chemical stability.^19–21^ However, PTFE is a classic antiadhesive material, which is deficient in compatibility.^22^ The apparent phase separation between physically mixed RDX, Al, and PTFE significantly damages its combustion performance. Dopamine is a strong adhesion of biological material, can spontaneously form a polymer under basic conditions, and has been demonstrated to adhere to virtually all types of inorganic and organic surfaces including PTFE.^9,23,24^ The core–shell particles can be prepared simply by self-polymerization of dopamine on the RDX surface in the component. The core/shell structured combines the unique characteristics of the core–shell material and is confirmed to be an effective strategy for synergistic performance.^23,25^ Meanwhile, the thickness can be well controlled by changing the polymerization time and monomer concentration to ensure that the obtained reactivity can be adjusted.^9^ In this study, RDX@PTFE–Al quasi-core/shell structured composites are prepared through interface control with polydopamine (PDA) as the interface layer, and the rapid combustion of PTFE/Al on the surface after ignition is performed for heat transfer to RDX. Concurrently, the high pressure and a large amount of gas generated by the combustion of RDX accelerate the mass and heat transfer of PTFE/Al as the shell.

## 2. Experimental section

### 2.1. Materials

RDX was synthesized in our institute (RDX >99%). The Al nanoparticles with an average diameter of 60 nm and an active metal content of 75 wt% determined by thermogravimetric analysis (TGA) were purchased from Shanghai Paddy Material Technology Co., Ltd, China. PTFE (200 nm) was procured from Zhongcheng Plastic raw materials Business Department, Dong Guan, China. Tris–hydrochloride buffer (pH = 8.5, 1 M) was obtained from Shanghai Aladdin Biochemical Technology Co., Ltd, China. 3-Hydroxytyramine hydrochloride (98%) was acquired from Shanghai Acmec Biochemical Co., Ltd, China. Ethanol was bought from Chengdu Cologne Chemicals Co., Ltd, China. Ultrapure water with a resistivity of 18.2 MΩ cm was produced UPC-I-10T apparatus (Chengdu Youpu Ultra Pure Technology Co., Ltd.).

### 2.2. Surface modification of RDX crystals

The PDA modification RDX was synthesized as follows. Tris solution (10 mM) was prepared, and the pH value was detected to be 8.5–8.6 (PHS-3CW microcomputer pH/mV meter, Bante, China), then dopamine was added to prepare a 0.4 g L^−1^ concentration solution. Afterward, the RDX underwent dopamine polymerization through immersion in the prepared dopamine solution, stirred at 500 RPM for 6 h. After vacuum filtration and washing with ultrapure water, the PDA modification RDX was obtained by being dried at 40 °C in a vacuum oven. The sample was denoted as pRDX.

### 2.3. Ultrasonic synthesis of pRDX@PTFE–Al composite

The experimental method of the quasi-core/shell structures was performed as follows. Firstly, 0.477 g of PTFE and 0.223 g of Al nanoparticles were dispersed in 20 mL ethanol and irradiated by high-intensity ultrasound for 5 min with an output sound power of 200 W and a frequency of 40 kHz (Model KQ5200DE, Kunshan Ultrasonic Instrument Co., Ltd.). Meanwhile, 1.4 g of pRDX was dispersed for 5 min under the same conditions. Subsequently, the ultrasonically dispersed pRDX crystals were immediately added to the above PTFE/Al suspension, and sonication was continued for 15 min. After vacuum filtration and washing with ultrapure water, the quasi-core/shell composite was obtained by being dried at 40 °C in a vacuum oven. The sample was denoted as pRDX@PTFE–Al. Additionally, physical mixtures of the same mass ratio were prepared as a reference, labeled RDX/PTFE/Al. The equivalence ratio of PTFE and Al was determined by reaction stoichiometric ratio based on equation: 3(CF_2_) + 2Al → 2AlF_3_ + 3C. Since the content of activated Al was about 75%, there was an excess of Al mass.

### 2.4. Characterization

The morphology of samples was characterized by scanning electron microscopy (SEM) measurements with an Ultra-55 microscope (Carl Zeiss, Germany). Fourier-transform infrared (FT-IR) spectra were acquired using a Bruker Tensor 27 (BRUKER, Germany) in 400–4000 cm^−1^. X-ray diffraction patterns (XRD, X Pert Pro, PANalytical B.V., Netherlands) were obtained on Bruker D8-Advance diffractometer equipment at Cu Kα radiation (*λ* = 0.15405 nm). The scan range, scan step size, and time per step for data collection are 3–80°, 0.03, and 10.16, respectively. X-ray photoelectron spectroscopy (XPS) was performed on a K-ALPHA + electron spectrometer (Thermo Fisher Scientific, USA) with Al Kα irradiation (1486.68 eV, 12 kV) to analyze the valence states of the elements. Besides, Differential scanning calorimeter (DSC) and thermogravimetric (TG) data were obtained using a STA449F5 Jupiter instrument (NETZSCH, China) with a Pt–Rh + Al_2_O_3_ crucible and 1.5 mg of samples. The measurements were conducted under a dynamic atmosphere of Ar at a flow rate of 60 mL min^−1^ with a heating rate of 20 °C min^−1^. The impact and friction sensitivities were examined on a BAM fall hammer BFH-12 and a BAM friction apparatus FSKM-10 (OZM Research, Czech Republic). The combustion process, energy output, and the interactions between the components are investigated using high-speed photography shoot open burning experiments. A nickel-chromium resistance wire (diameter: 0.25 mm) was used for heating ignition at one end of the suspension. Combustion velocity testing: the pRDX@PTFE–Al composite sample are ignited by Cr–Ni alloy silk (diameter: 0.4 mm, length: 3 cm, melting point: 1200 °C) in the open air, which is heated by direct current power supply (MS-3010D, MAISHENG) under 7.5 V. The combustion rate and combustion process of the samples were tested using high speed photography (Canon, EOSKiss70D).

## 3. Results and discussion

### 3.1. Morphology and chemical composition

**Fig. 1:**
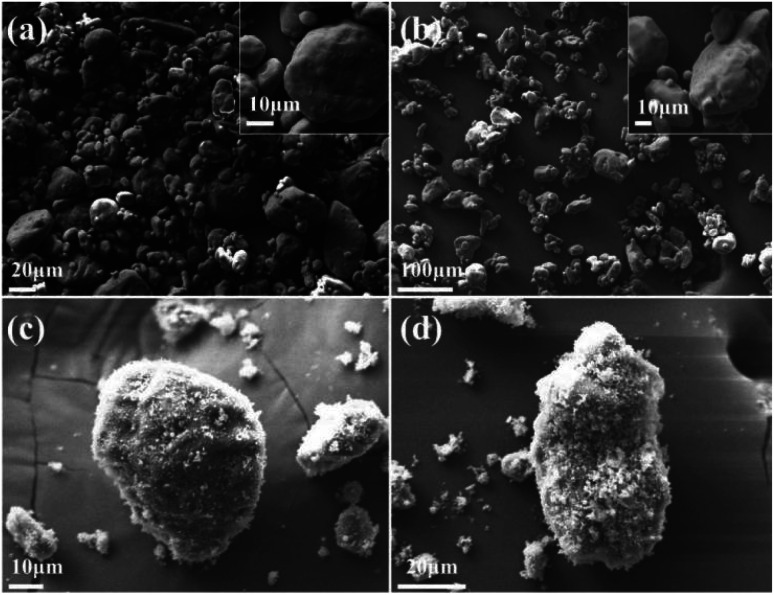
SEM images of (a) raw RDX; (b) pRDX; (c and d) pRDX@PTFE–Al.

Fig. 1 illustrates the SEM images of the raw RDX, pRDX, and pRDX@PTFE–Al. Since the raw RDX has not undergone recrystallization, the particle size distribution was not uniform (Fig. 1a). PDA is used as a surface modification tool to modify particles in Tris–HCl solution with pH = 8.0–8.5 and dopamine concentration of 2 g L^−1^ by a simple dip-coating process.^26–28^ PDA is employed to modify ammonium nitrate explosives and nitro explosives. The initial concentration of the dopamine solution is 2 g L^−1^. It is continuously stirred for 6 h under an air atmosphere. The content of PDA in this core/shell structured is about 2.8 wt%,^29,30^ and the shell thickness exceeds 50 nm. However, dopamine concentration is an essential tool in controlling deposition kinetics and roughness of surfaces. In this experiment, a low concentration of dopamine (0.4 g L^−1^) was used for functionalized RDX crystals. From one perspective, a low concentration of dopamine is beneficial to reducing the formation of PDA particles and decreasing roughness. From another perspective, the surface energy of PDA is independent of dopamine concentration and does not affect the formation of quasi-core/shell composites.^31,32^

The high-resolution SEM image in Fig. 1b demonstrates the low roughness of the pRDX surface. The attachment of some of the smaller pRDX particles to the larger pRDX surface reveals the successful modification of the RDX crystal by PDA. Fig. 1c and d illuminates that PTFE/Al is more completely coated on the surface of pRDX particles of different sizes forming a quasi-core/shell structured composite. This verifies that the agglomerates are dispersed after ultrasonic dispersion. Fig. S1† exhibits the PDA-modified RDX for 3 h and 9 h, as well as the prepared composites under the same fabrication process. It is unveiled that the agglomeration phenomenon is insignificant when the PDA-modified RDX crystals for 3 h; nonetheless, the pRDX is covered to a lesser extent in the prepared composites, and the PDA-modified crystals for 9 h generate huge agglomerates and are not covered by PTFE/Al to a particularly huge extent compared to pRDX@PTFE–Al.

**Fig. 2:**
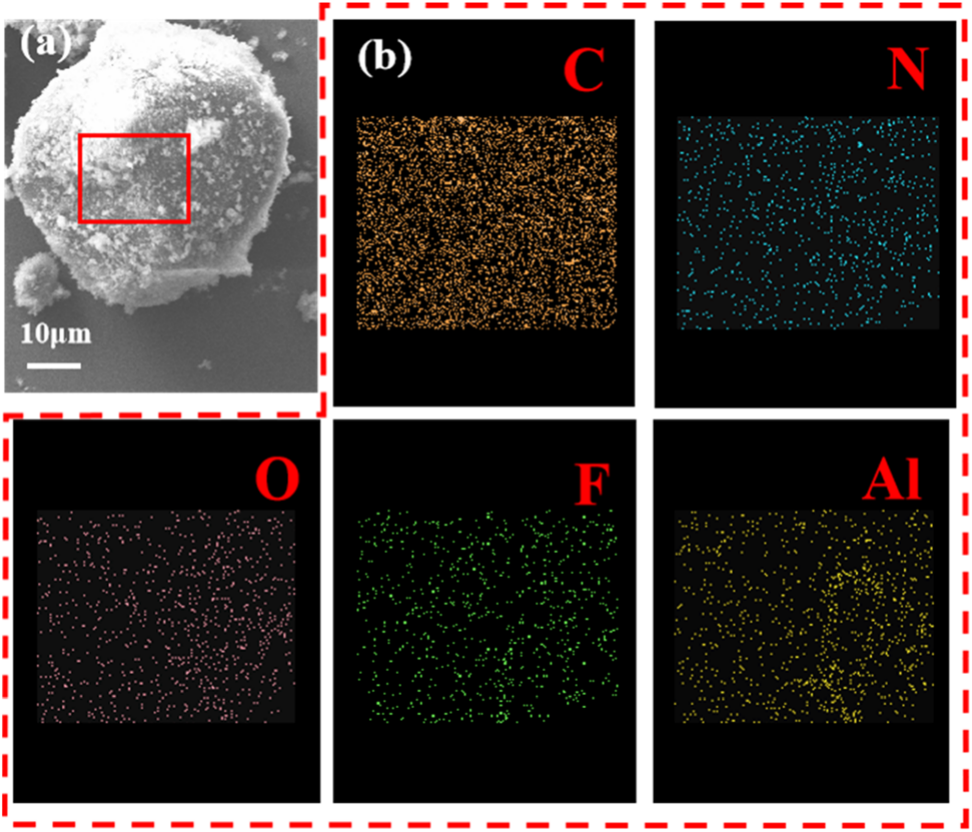
(a) EDS image area of pRDX@PTFE–Al sample; (b) EDS images of C, V, O, F and Al.

Fig. 2 illustrates EDS images of pRDX@PTFE–Al sample. The results of the EDS energy spectrum scan by the surface of the composite sample surface are shown in figure. It can be clearly found that the surface of the composite sample contains five elements of C, N, O, F and Al. It can be tentatively concluded that Al and PTFE are coated on the surface of RDX under the action of PDA.

**Fig. 3:**
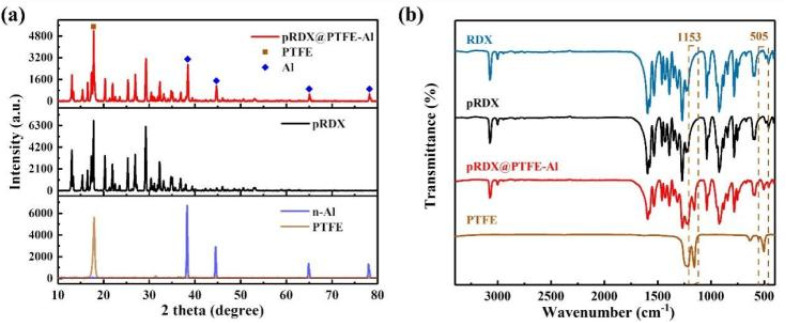
(a) XRD patterns of pRDX@PTFE–Al, pRDX, Al, and PTFE. (b) FT-IR spectra of the raw RDX, pRDX, pRDX@PTFE–Al, and PTFE powder in the range of 400–3400 cm^−1^.

Fig. 3a depicts the XRD spectrum of the raw material and pRDX@PTFE–Al. The characteristic diffraction peaks of RDX modified by PDA are consistent with those of JCPDS database (PDF 00-046-1606). The results are shown in Fig. 3a. The characteristic diffraction peaks of the prepared composites correspond to the original RDX particles, reflecting that the process of preparing quasi-core/shell structured composites does not change the crystal morphology of RDX. The characteristic diffraction peaks of Al are presented in the composite samples, and the characteristic diffraction peaks of PTFE are not observed because the characteristic peaks at 17.9° coincide with RDX. The characteristic chemical bonds of RDX, pRDX, and pRDX@PTFE–Al were determined to be in the range of 400–3400 cm^−1^ through FT-IR analysis. As suggested in Fig. 3b, the existence of the characteristic peak of RDX is noticeable.^33^ Additionally, characteristic functional groups or chemical bonds do not recognize the PDA in the modified RDX owing to the low content of the PDA coating and the similarity between chemical elements and chemical bonds with energy-containing crystals. The composite exhibits a new characteristic peak at 505 cm^−1^ and 1153 cm^−1^ under the stretching vibration of the C–F bond,^19,22^ validating the presence of PTFE. The design of a quasi-core/shell structure with PTFE/Al adhered to the pRDX surface is achieved based on the above analysis and the SEM images.

**Fig. 4:**
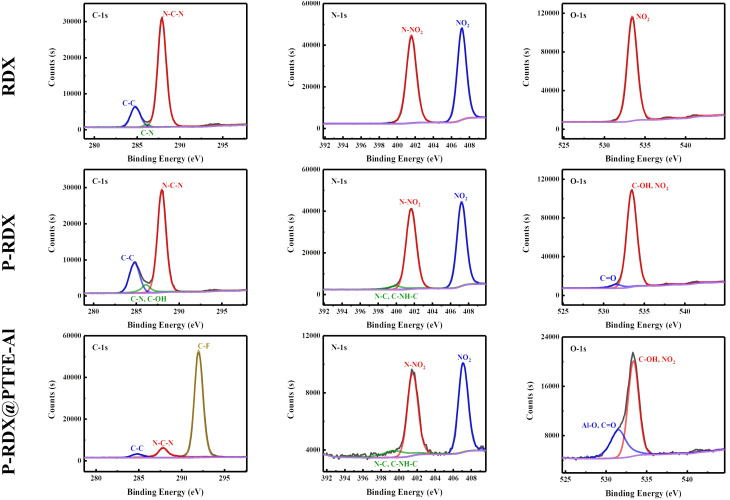
High-resolution XPS spectra of RDX, pRDX, and pRDX@PTFE–Al.

The structure of RDX, pRDX, and pRDX@PTFE–Al in the quasi-core/shell structure is better understood using X-ray photoelectron spectroscopy (Fig. 4). Fig. S2 and S3† presents the full spectra of the samples in ESI† and the high-resolution XPS spectra of Al and F elements. Table S1† provides the surface elemental composition of RDX, pRDX, and pRDX@PTFE–Al, as determined by XPS. The C 1s spectrum was deconstructed into three peaks corresponding to the C–C bond (284.80 eV), C–N–H bond (286.14 eV), and N–C–N bond (287.88 eV) in RDX.^34^ Notably, the peak intensity of C–N/C–O increases at 286.12 eV, and the N/C ratio of pRDX decreases from 0.77 of RDX to 0.66, confirming the successful coating of PDA on the surface of RDX crystals. In pRDX, the N1s spectrum exhibits two sharp peaks at 407.24 eV and 401.63 eV, corresponding to NO_2_ and N–NO_2_ binding energies typical of RDX, respectively.^35^ Besides, the peak at 399.93 eV was derived from the N–C bond.^34^ The oxygen O 1s spectrum reveals binding energies of 533.42 eV (NO_2_) for bonds at the surface of the RDX. The O 1s profile of pRDX is fitted into two peaks at 533.48 eV and 531.57 eV, which are assigned to the NO_2_ in RDX and C=O and unoxidized hydroxyl group in PDA, respectively.^36^ After modification with PDA, the basic peak pattern of pRDX powder is similar to that of RDX and can be regarded as a combination of RDX and PDA. In the composite material, the existence of the (–CF_2_–CF_2_–)_*n*_ bond at 292.01 eV proves that PTFE is successfully coated on the surface of pRDX. Compared with pRDX, the N 1s of the composite material have no change except for the significant reduction in strength. As demonstrated by comparing the O 1s peak of the composite and pRDX, the composite has a significant Al–O peak at 531.52 eV. This is induced by the aluminum oxide layer on the surface of the n-Al, which also validates that the n-Al is present in the composite.

### 3.2. Thermal analysis of pRDX@PTFE–Al

**Fig. 5:**
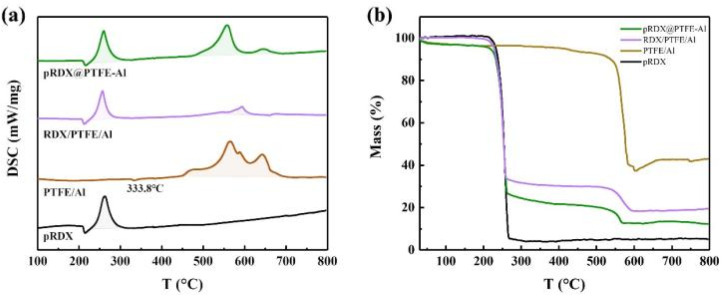
(a) DSC curves of the pRDX@PTFE–Al, RDX/PTFE/Al, PTFE/Al, and pRDX in the argon atmosphere; (b) TG curves of the pRDX@PTFE–Al, RDX/PTFE/Al, PTFE/Al, and pRDX in the argon atmosphere.

The thermal properties of the as-prepared pRDX@PTFE–Al composite are investigated using DSC-TG. The performance in an Ar atmosphere at 20 °C min^−1^ heating rate conditions is summarized in Fig. 5. Instruments Universal Analysis software was adopted to determine the energy output. In Table 1, the exothermic process of the sample is divided into two stages: RDX and PTFE/Al, which illustrate the changes occurring during the heating process of the samples.

**Table 1:** Summary of DSC-TG data of pRDX, PTFE/Al, RDX/PTFE/Al, and pRDX@PTFE–Al

Samples	First stage		Second stage	
	Exothermic peak (°C)	Heat release of exotherm (J g^−1^)	Exothermic peak (°C)	Heat release of exotherm (J g^−1^)
pRDX	262.5	1308.0	—	—
PTFE/Al	—	—	565.2	5477.0
RDX/PTFE/Al	257.0	855.6	593.8	401.5
pRDX@PTFE–Al	259.9	1171.0	558.2	2498.0

The TG curve of the composite material presents a significant multistep weightlessness process caused by the decomposition of RDX and PTFE/Al. In Fig. 5b, mass loss recorded pRDX and PTFE/Al powder was 94.8% and 57.0%, respectively. The mass loss of pRDX@PTFE–Al decreases slowly in the initial stage of weight loss attributed to the release of adsorbed gas or moisture from the surface of the sample.^37^ The mass loss of the sample in the first stage and the second stage is 74.9% and 12.9%, respectively. Correspondingly, the exothermic process of the composite material can be divided into two portions. The intense exotherm before 300 °C in the tested samples can be ascribed to RDX decomposition, which corresponds to the mass loss in the TG curve of the samples containing RDX in Fig. 5b. The second exothermic stage is associated with the decomposition reaction between PTFE and Al. The small endothermic peak at 333.8 °C in the pure PTFE/Al sample is related to the melting of PTFE, and a slight exothermic peak around 450 °C can be derived from the pre-ignition reaction (PIR) between PTFE and Al_2_O_3_ passivation layer.^14^ Notably, the second-stage reaction in the RDX/PTFE/Al sample is not significant due to the physical dispersion inhomogeneity, and the total sample heat release is significantly lower. In contrast, the composite pRDX@PTFE–Al exhibited the superiority of quasi-core/shell structure. The maximum decomposition temperature of pRDX is reduced by 2.6 °C, the exotherm is more concentrated in the second stage and the exotherm peak is advanced by about 7.0 °C. Moreover, the total exotherm is significantly improved relative to pRDX.

### 3.3. Combustion performance

**Fig. 6:**
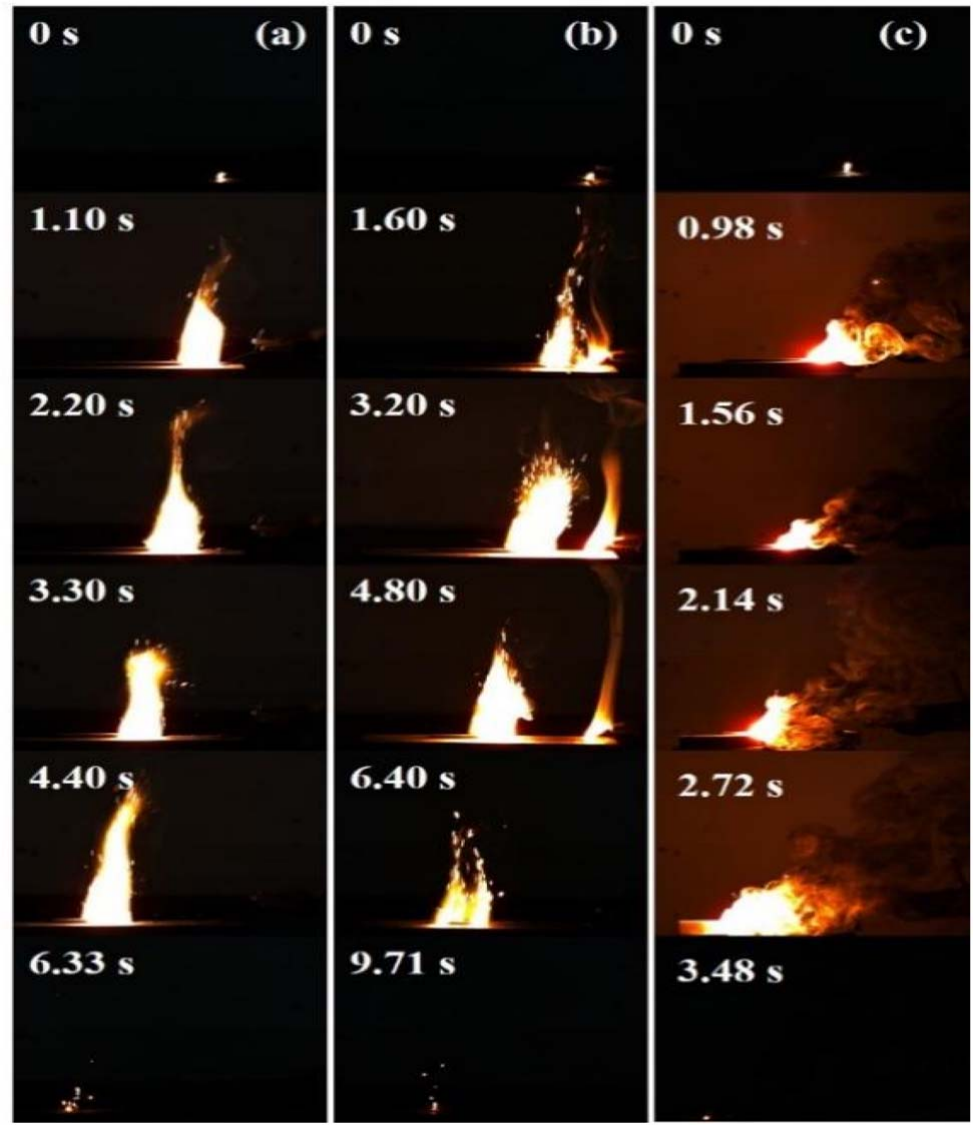
(a) pRDX@PTFE–Al; (b) RDX/PTFE/Al; (c) PTFE/Al.

Considering the slow heating rate in the thermal analysis, the main reactions of each component in the test samples are independent of each other. Fig. 6 illustrates the high-speed video flame images during the electric ignition experiments of pRDX@PTFE–Al, RDX/PTFE/Al, and PTFE/Al, with a mass of 0.4 g. The length of the grain is 80 mm (the error is less than 2 mm), and the width and height are 2 mm. The raw RDX sample reacts at the contact part of the resistance wire to produce yellow combustion products, and no self-propagating combustion is observed. As suggested in Fig. 6, the recording time of the first occurrence of the spark is 0 s. Under the same test conditions, RDX/PTFE/Al ends combustion after 9.71 s, with a sustained combustion time much greater than that of pRDX@PTFE–Al at 6.33 s.

The PTFE/Al sample caught fire immediately after heating and could maintain self-propagating combustion. A large amount of smoke was observed accompanied by a strong flame generation. Regarding the physically mixed sample RDX/PTFE–Al, the addition of MIC caused the sample to burn vigorously, while the combustion transfer process was unstable with flame dispersion and sparks splashing into the air. Thus, it was not favorable for practical applications. In addition to incomplete combustion leading to reduced energy output, some splashed solid particles and instability of flame propagation may also interrupt the propagation of the reaction, resulting in the inability to provide a continuous energy output and weakening the reliability of the high-energy system. In contrast, the pRDX@PTFE–Al burns faster, and the flame propagation is more stable, allowing for more reliable energy output. Specifically, it can be attributed to the formation of a homogeneous PDA coating on the RDX surface through a simple *in situ* polymerization, which assures good contact between the components, especially the lack of enhanced compatibility with PTFE, and thus contributes to a facilitated quality and heat transfer process.

Besides, the RDX core and the PTFE/Al on the surface play complementary roles in the combustion. The MIC adhering to the surface of pRDX guarantees that the sample can sustain self-propagating combustion, while RDX, as a good gas generator, releases a large amount of gas during rapid decomposition, which not only retards the sintering of Al but also promotes the heat transfer dominated by convection to a certain extent. The interaction between the two ensures a stable propagation of the flame. The reduced combustion rate of the composite relative to PTFE/Al is provoked by the release of associated gases to escape from the reaction zone.^38^ A large amount of nitrogen, carbon dioxide, and aluminum fluoride sublimated in the thermite reaction produced by RDX as the main component of the samples in an unconstrained combustion environment escapes into the environment instead of accelerating the flame front, reduces the energy output rate, fails to assist in the reaction and the convective mode of energy propagation, and thus weakens the combustion.^11^

A small amount of condensed combustion products was characterized and analyzed using SEM, as presented in Fig. S4.† In addition to the disordered and inhomogeneous combustion products, some large particles are in the shape of regular spheres and covered by small and loose nanoparticles (Fig. S4a–c†). The Fig. S4d† images of the raw Al imply that these large particles are larger size Al, which did not react completely during the combustion process. Additionally, the surface should be covered by PTFE that was molten and recondensed as well as AlF_3_ generated by combustion. In conclusion, the quasi-core/shell structure and reaction characteristics of pRDX@PTFE–Al enhance the energy release performance of the entire high-energy system and provide continuous flame propagation and stable energy output. From this perspective, its practical application is more reliable. The combined experimental results of thermal and combustion analyses reflect that the composite enhances the interfacial contact between the components, improves the heat feedback, and considerably enhances the combustion performance.

### 3.4. Mechanical sensitivities of the raw RDX and as prepared pRDX@PTFE–Al particles

The results of friction and impact sensitivities of the RDX, pRDX, RDX/PTFE/Al, and pRDX@PTFE–Al are summarized in Table 2.

**Table 2:** Friction and impact sensitivities of the RDX and pRDX@PTFE–Al particles

Samples	Friction sensitivities (N)	Impact sensitivities (J)
RDX	240	25
pRDX	296	31
RDX/PTFE/Al	80	2
pRDX@PTFE–Al	288	27.5

Experiment results on a physical mixed sample uncover that the addition of MIC while providing a high energy density and reaction rate can exhibit an extremely high mechanical sensitivity. In addition to hot spot growth caused by crystal defects during impact and adiabatic compression, the presence of metallic aluminum concentrates the stress in a small area or at a point, causing additional hot spot growth and increasing the impact sensitivity. Nonetheless, the soft coating on the surface lessens the hot spot formation by absorbing the impact energy, and the introduction of heat-absorbing materials mitigates the hot spot formation during the impact.^39^ This is the reason for the introduction of PDA coating. Specifically, PDA has been used more in the field of desensitization of energetic materials.^28,29,39^ Moreover, PTFE can be coated on the surface of the coating and is effective as a good lubricant in improving the frictional properties of the composite.^22,40^ It can curtail the deformation between the explosive and the rigid surface, reducing the mechanical properties of the composite. Thus, the surface of RDX is successfully coated with PTFE, which has a certain buffering effect under the impact of falling darts, effectively slowing down the formation of hot spots. So the susceptibility of the surface components of the composite sample is lower than that of the original RDX, and in the process of impact susceptibility test, the falling hammer first comes into contact with the low-sensitivity components on the surface, which has a certain buffering effect on the impact force. The results implied that the mechanical sensitivity of the quasi-core/shell structure was weakened with a good desensitizing effect.

## 4. Conclusion

In this paper, pRDX@PTFE–Al quasi-core/shell structured high-energy explosives were prepared by ultrasonic dispersion method using the proper introduction of PDA as a surface modifier. With the advantage of the structure, the own characteristics of each component of the system were maintained, and composites with better combustion properties and safety were prepared. This study confirmed that the integration of homogeneous and inhomogeneous materials in energetic materials was an effective complementary strategy to improve the performance of the overall energy system. Further research is required for practical applications.

## Conflicts of interest

There are no conflicts to declare.

## Supplementary Material

RA-013-D3RA02732E-s001
