# Supplementary material for: Preparation of quasi-core/shell structured composite energetic materials to improve combustion performance
Source: RSC Adv. 2023 Jun 13;13(26):17834–41. doi: 10.1039/d3ra02732e (PMC10262013; doi:10.1039/d3ra02732e)
Supplement: RA-013-D3RA02732E-s001 [file RA-013-D3RA02732E-s001.pdf]

Electronic Supplementary Material (ESI) for RSC Advances.
This journal is © The Royal Society of Chemistry 2023

Supporting information

Preparation of quasi-core/shell structured composite energetic materials to improve the combustion performance

Ruihao Wang*, Lanting Yang, Zhenwei Zhang, Wenkui Song, Dunju Wang，Changping Guo

Co-Innovation Center for New Energetic Materials, Southwest University of Science and Technology, Mianyang 621010, P. R. China.

Corresponding Author

* E-mail: ruihao0847@163.co m (Ruihao Wang)

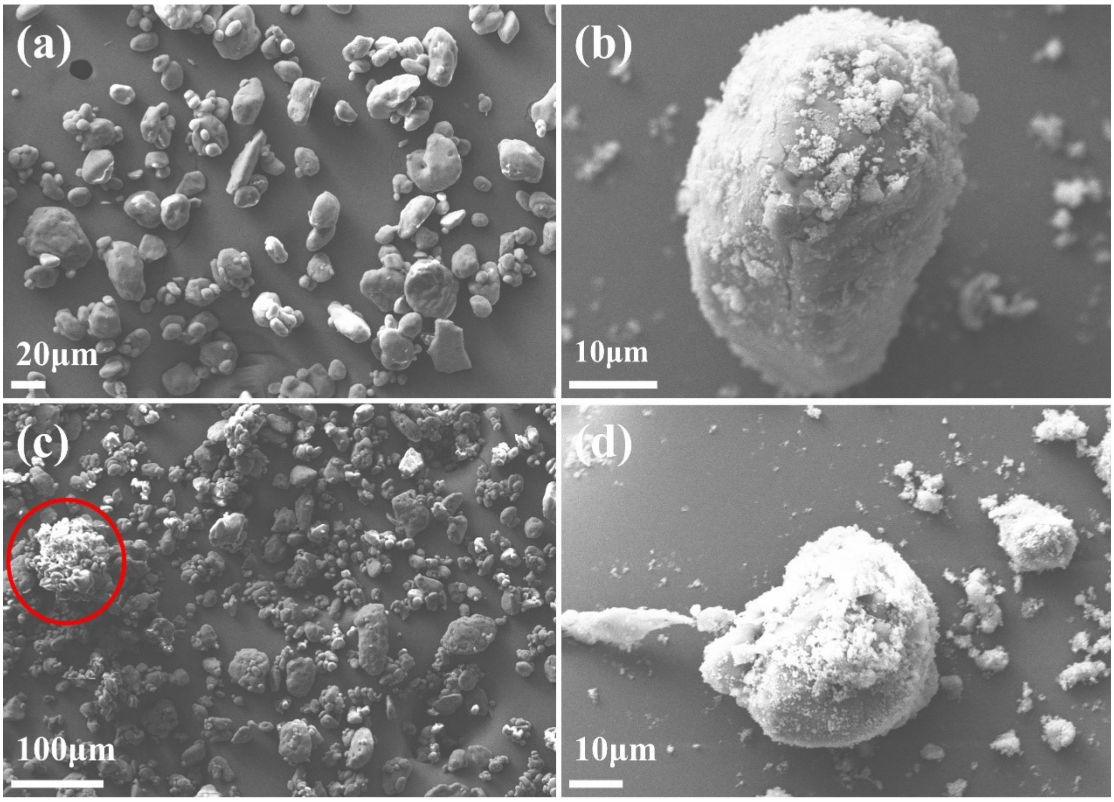

Fig. S1. SEM images of (a) pRDX-3h; (b) pRDX -3h@PTFE-Al; (c) pRDX -9h; (d) pRDX -9h@PTFE-Al

Table S1

Surface element composition of RDX, pRDX, and pRDX@PTFE-Al, as determined by XPS

Samples	C-1s (%)	N-1s (%)	O-1s (%)	Al-2p (%)	F-2p (%)	N/C
RDX	32.11	24.64	43.26	-	-	0.77
pRDX	34.76	23.66	41.58	-	-	0.68
pRDX@PTFE-Al	29.13	2.5	5.51	2.07	60.79	0.09

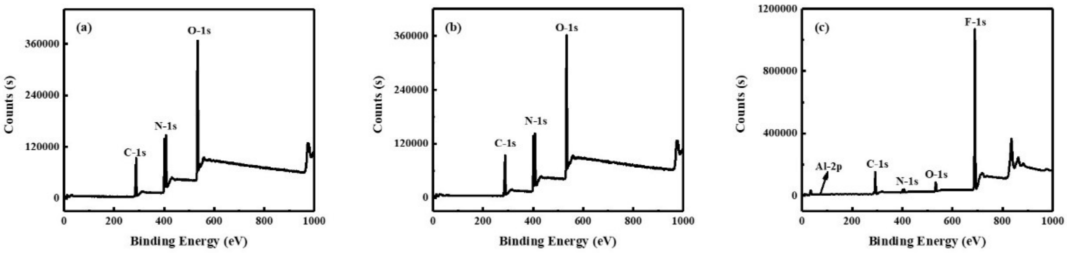

Fig. S2. XPS spectra of (a) raw RDX; (b) pRDX; (c) pRDX@PTFE-Al.

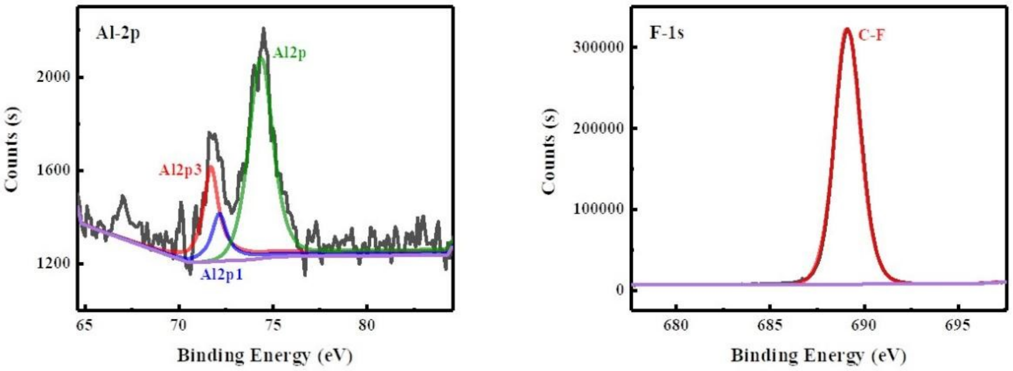

Fig. S3. High-resolution XPS spectra of Al and F elements in pRDX@PTFE-Al.

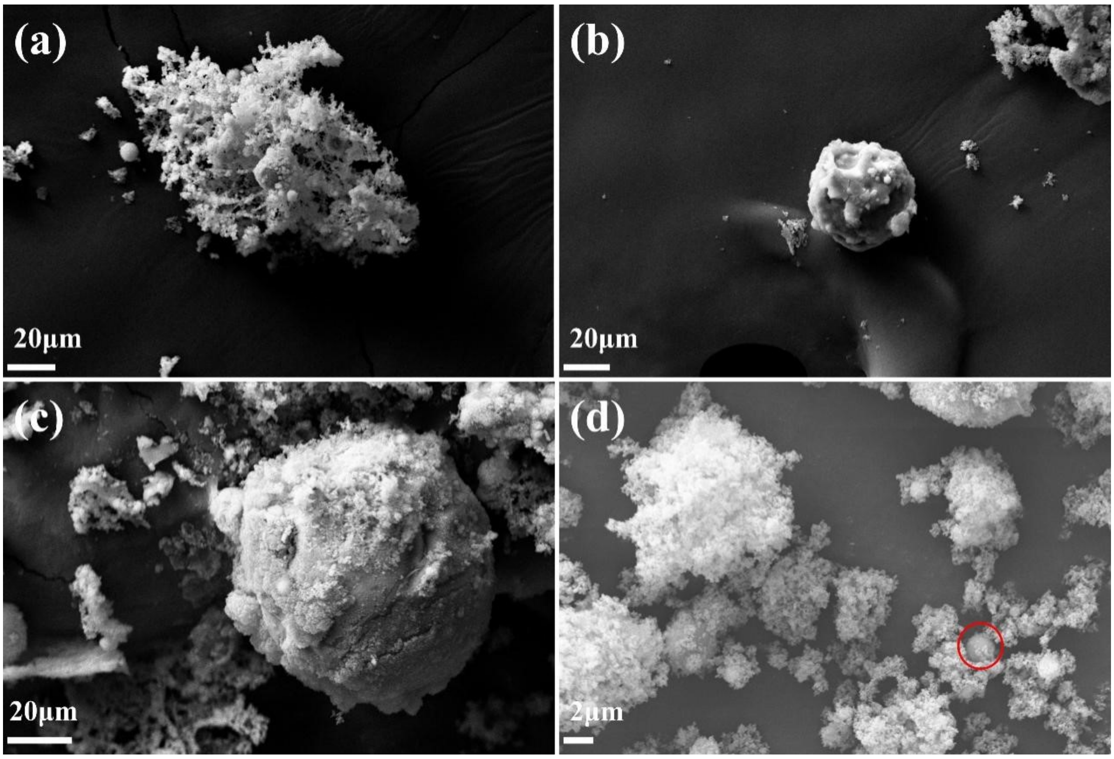

Fig. S4. SEM images of (a) (b) (c) combustion products; (d) raw Nano Al.
